# Cerebrospinal fluid circulating tumor cells as a quantifiable measurement of leptomeningeal metastases in patients with HER2 positive cancer

**DOI:** 10.1007/s11060-020-03555-z

**Published:** 2020-06-06

**Authors:** Rachna Malani, Martin Fleisher, Priya Kumthekar, Xuling Lin, Antonio Omuro, Morris D. Groves, Nancy U. Lin, Michelle Melisko, Andrew B. Lassman, Suriya Jeyapalan, Andrew Seidman, Anna Skakodub, Adrienne Boire, Lisa M. DeAngelis, Marc Rosenblum, Jeffrey Raizer, Elena Pentsova

**Affiliations:** 1 Department of Neurology, Memorial Sloan Kettering Cancer Center, 1275 York Avenue, New York, NY 10065 USA; 2 Department of Laboratory Medicine, Memorial Sloan Kettering Cancer Center, New York, NY 10065 USA; 3 Present Address: Department of Neurology, National Neuroscience Institute, Singapore, 308433 Singapore; 4 Present Address: Department of Neurology, Yale University, New Haven, CT 06511 USA; 5 Department of Neurology, Texas Oncology, Austin, TX 78705 USA; 6 Department of Neurology, Dana Farber Cancer Institute, Boston, MA 02215 USA; 7 Department of Medicine, UCSF, San Francisco, CA 94143 USA; 8 Department of Neurology and Herbert Irving Comprehensive Cancer Center, New-York Presbyterian Hospital/Columbia University Irving Medical Center, New York, NY 10032 USA; 9 Department of Neurology, Tufts University, Medford, MA 02155 USA; 10 Department of Pathology, Memorial Sloan Kettering Cancer Center, New York, NY 10065 USA; 11 Department of Medicine, Memorial Sloan Kettering Cancer Center, New York, NY 10065 USA; 12 Department of Neurology, Northwestern University, Evanston, IL 60208 USA

**Keywords:** Leptomeningeal metastases, Cerebrospinal fluid, Circulating tumor cells, Biomarker, Liquid biomarkers

## Abstract

**Purpose:**

The CellSearch® system has been used to identify circulating tumor cells (CTCs) in cerebrospinal fluid (CSF) to diagnose leptomeningeal metastasis (LM) in patients with epithelial cancers. Using this system, we prospectively explored sequential CSF CTC enumeration in patients with LM from HER2+ cancers receiving intrathecal (IT) trastuzumab to capture dynamic changes in CSF CTC enumeration.

**Methods:**

CSF from patients enrolled in an IRB-approved phase I/II dose escalation trial of IT trastuzumab for LM in HER2+ cancer (NCT01325207) was obtained on day 1 of each cycle and was evaluated by the CellSearch® platform for CTC enumeration. The results were correlated with CSF cytology from the same sample, along with clinical and radiographic response.

**Results:**

Fifteen out of 34 patients with HER2+ LM were enrolled in CSF CTC analysis; 14 were women. Radiographic LM was documented in 14 (93%) patients; CSF cytology was positive in 6 (40%) and CSF CTCs were identified in 13 (87%). Median CSF CTC was 22 CTCs (range 0–200 +) per 3 ml. HER2/*neu* expression analysis of CTCs was performed in 8 patients; 75% had confirmed expression of HER2*/neu* positivity in CSF and HER2/*neu* expression was absent in 25%. Four of 10 patients received 7 or more cycles of IT trastuzumab; in 3 of these patients, increase in CSF CTCs enumeration from baseline was detected 2–3 months prior to changes seen on MRI, and while CSF cytology remained negative.

**Conclusion:**

Our study demonstrates that enumeration of CSF CTCs may provide dynamic, quantitative assessment of tumor burden in the central nervous system compartment during treatment for LM and prior to changes on MRI or CSF cytology.

**Trial Registration:** Clinicaltrials.gov: NCT01325207; registered March 29th, 2011.

**Electronic supplementary material:**

The online version of this article (10.1007/s11060-020-03555-z) contains supplementary material, which is available to authorized users.

## Introduction

Diagnosis, prognosis, and treatment of leptomeningeal metastasis (LM) carries a significant implication for oncologists and patients [1–3]. Diagnosis and management currently relies on a combination of magnetic resonance imaging (MRI) and cerebrospinal fluid (CSF) cytology [1, 2, 4], which are two tools with low sensitivity in the early stages of LM [2, 3]. Moreover, once the diagnosis is established, MRI and CSF cytology are insensitive to determine treatment response [1, 4]. To overcome these limitations, a variety of techniques including circulating tumor cell (CTC) [5–8] technologies have been developed, adapted to CSF, and validated. This includes CSF CTC methodology by the CellSearch® system (Menarini Silicon Biosystems), which is a highly-sensitive method to diagnose LM with better diagnostic performance compared to CSF cytology and MRI [9–12]. The value of CSF CTCs as a prognostic biomarker, and as a biomarker of therapeutic response, has not yet been elucidated in clinical trials; however, it is well established in plasma [5, 8].

Major clinical societies have been working to establish guidelines for a unified approach to diagnose and manage LM. The current European Association of Neuro-Oncology-European Society for Medical Oncology (EANO-ESMO) guidelines provide recommendations regarding the diagnosis and treatment of LM from solid and liquid cancers [13], which include MRI of the brain and spine in conjunction with CSF cytologic analysis. Similarly, the Leptomeningeal Assessment in Neuro-Oncology (LANO) working group (a part of the Response Assessment in Neuro-Oncology [RANO] group) recommends establishing a diagnosis of LM based on a combination of symptom constellation, brain and spine MRIs, and CSF cytology [14, 15]. However, MRI findings can be non-diagnostic, particularly at early stages, and definitive findings may therefore appear only in later stages. Likewise, while positive CSF cytology represents the gold standard for diagnosing LM, its initial sensitivity is quite modest, and repeated CSF samples may be required to increase sensitivity to about 90% [2, 3].

The EANO-ESMO guidelines reference several promising new platforms to improve sensitivity for tumor cell detection in the CSF. The LANO group proposes to include CSF CTCs, as well as other platforms that allow for molecular profiling such as CSF circulating tumor DNA. Importantly, it also states prospective studies must include evaluation of liquid biopsies [16].

Further work is needed to establish the clinical utility of these analyses as biomarkers of both disease diagnosis and response to treatment, as well as to incorporate the use of CSF CTCs into clinical practice. The goal of this study was to evaluate the value of CSF CTCs as biomarkers prior to and during treatment in patients with LM from HER2+ cancers who were receiving treatment with intrathecal (IT) trastuzumab on a clinical trial [17]. We used epithelial cell adhesion molecule (EpCAM)-based rare cell capture technology (RCCT), which utilizes an immunomagnetic CTC selection method based on EpCAM antibody conjugated ferroparticles and is a sensitive technique to detect LM from epithelial tumors [10–12, 18, 19]. We found that CSF CTC enumeration effectively capture at diagnosis, and may provide quantifiable measurement of tumor burden in a disease setting such LM, which is notoriously difficult to quantify and may potentially serve as a biomarker of treatment response.

## Methods

**Fig. 1:**
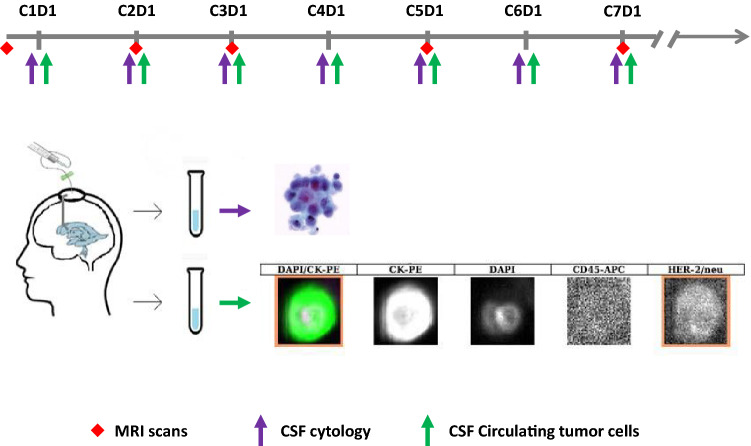
Schema of the CSF and MRI assessment and CSF tests workflow indicating that CSF was collected via an Ommaya reservoir prior to each cycle of treatment with intrathecal trastuzumab. Then 3–5 ml of fluid were processed for CSF cytology (top panel) and another 3.5 ml of fluid for CSF circulating tumor cells (bottom panel). CTCs in the CSF were identified as CK-PE+, DAPI+ and CD45−. HER2/neu expression analysis of CTCs was performed and shown in the last channel

This multi-institutional phase I/II dose escalation trial of IT trastuzumab for LM [17] was conducted in six medical centers from August 2012 to July 2016, and included CSF CTC enumeration as an optional exploratory arm to define whether CSF CTCs were present in the CNS compartment at trial entry and whether there was a quantitative change in CTCs during treatment. CSF CTC enumeration was correlated with CSF cytology collected at the same time prior to drug instillation on day 1 of each cycle, and it was correlated with neuroimaging performed at baseline, prior to initiation of cycle 2, cycle 3, and then prior to every odd cycle thereafter (up to 3 days prior to initiation of the next cycle) (Fig. 1). LM was defined by neuroimaging demonstrating contrast enhancement in the usual LM pattern and/or positive CSF cytology. Overexpression of the human epidermal growth factor receptor 2 (HER22/neu) was confirmed by either immunohistochemical staining of 3+ or greater, or by fluorescence in situ hybridization (FISH) analysis of the primary or metastatic tissue.

Patients had an intraventricular reservoir (Ommaya) placed prior to initiation of IT therapy. IT trastuzumab was administered via an Ommaya reservoir twice weekly for cycle 1 (1 cycle = 28 days), then once a week for cycle 2 and every 2 weeks thereafter for subsequent cycles. CSF for cytology, as well as for CTC enumeration, was obtained immediately prior to drug instillation on day 1 of each cycle (Fig. 1). Three milliliters of CSF were transferred to a CellSave preservative tube and delivered from multiple participating institutions within 24–72 h from the CSF collection to Memorial Sloan Kettering Cancer Center, where the CSF CTC analysis was performed. MRIs of the brain and spine were performed prior to cycles 2 and 3, and then prior to every other cycle while patients remained on study. Progression of disease (POD) was defined as definite or probable regardless of the CSF cytology when new areas of leptomeningeal enhancement on MRI scan were seen and/or definite clinical POD secondary to LM regardless of the radiographic results.

The CellSearch® (Menarini Silicon Biosystems) platform was used to enumerate CTCs in CSF specimens. The CellSearch® Assay is an immunomagnetic isolation technique using antibodies to EpCAM conjugated to magnetic beads for the immunomagnetic capture of EpCAM-expressing epithelial cells. A CTC was defined as a cell that expresses cytokeratin (CK), displays a nucleus when stained with 4,6-diamidino-2-phenylindole (DAPI), and has no expression of CD45. CSF CTCs were also evaluated for HER2/*neu* expression using a specific Her2/*neu* antibody phenotyping marker reagent (Menarini Silicon Biosystems, cat#LN7900006). The Her2/*neu* CTC assay is not FDA approved, but rigorous performance testing was performed in accordance with Clinical Laboratory Improvement Amendments (CLIA) regulatory requirements and adapted for use on the CellSearch® platform. We were able to identify Her2/*neu* Ab+, CK-PE+, DAPI+ and CD45- cells using the CellSearch® method. Results were reported as number of CTCs per 3 ml of CSF (CSF CTCs/3 ml). The enumeration of CSF CTCs was limited by standard approach to 200+ CSF CTCs/3 ml though even higher numbers of CTCs were seen. This study was approved after review by the Institutional Review Boards and was conducted in accordance with the Declaration of Helsinki and International Conference on Harmonization Good Clinical Practice. All patients provided written informed consent before enrolment.

## Results

Fifteen out of 34 patients were enrolled in the CSF CTC analysis: 14 patients were women with breast cancer and 1 was a man with colon cancer. At trial entry, positive CSF cytology was confirmed in 6 patients and suspicious in 4 (Table 1). Fourteen patients had radiographic evidence of LM. CSF CTCs were identified in 13/15 (87%) patients; median CSF CTC enumeration was 22 CSF CTCs/3 ml (range 1–200+/3 ml). CSF CTCs were detected in 4 patients with negative CSF cytology. Two patients, 1 with negative and 1 with suspicious CSF cytology, had no detectable CSF CTCs. The Her2/*neu* CTC assay was performed in 8/15 patients with detectable CTCs, and Her2/*neu* expression of CTCs was confirmed in 6/8 (75%).

**Table 1:** Baseline clinical characteristics, CSF cytology, CSF circulating tumor cells, and MRI results of patients

Patient	Diagnosis	LM on MRI	Site of LM on MRI	CSF cytology	CSF CTCs/3 ml	CSF CTC HER2 expression
1	Breast Ca	+	B	Suspicious	0	NA
2	Breast Ca	+	B	Negative	0	NA
3	Breast Ca	+	B	Negative	1	Absent
4	Breast Ca	+	B + S	Suspicious	3*	Present
5	Breast Ca	+	B	Negative	5	Present
6	Breast Ca	+	B + S	Negative	7*	Present
7	Breast Ca	+	B	Negative	10	Present
8	Breast Ca	+	S	Positive	22	Present
9	Colon Ca	+	S	Suspicious	81	Absent
10	Breast Ca	+	S	Suspicious	139	x
11	Breast Ca	+	B + S	Positive	146	x
12	Breast Ca	+	S	Positive	166*	x
13	Breast Ca	+	B	Positive	200+	x
14	Breast Ca	+	B	Positive	200+*	Present
15	Breast Ca	−	na	Positive	200+*	x

*ca* cancer, + positive, − negative, *s* suspicious, *x* not performed, *lm* leptomeningeal metastases, *mri* magnetic resonance imaging, *B* brain, *S* spine, *csf* cerebrospinal fluid, *ctc* circulating tumor cell, *ml* milliliter, *HER2+ *human epidermal growth factor 2, *r* radiographic, *na* not applicable, *x* not done

*Patients came of study as they did not complete cycle 1

**Fig. 2:**
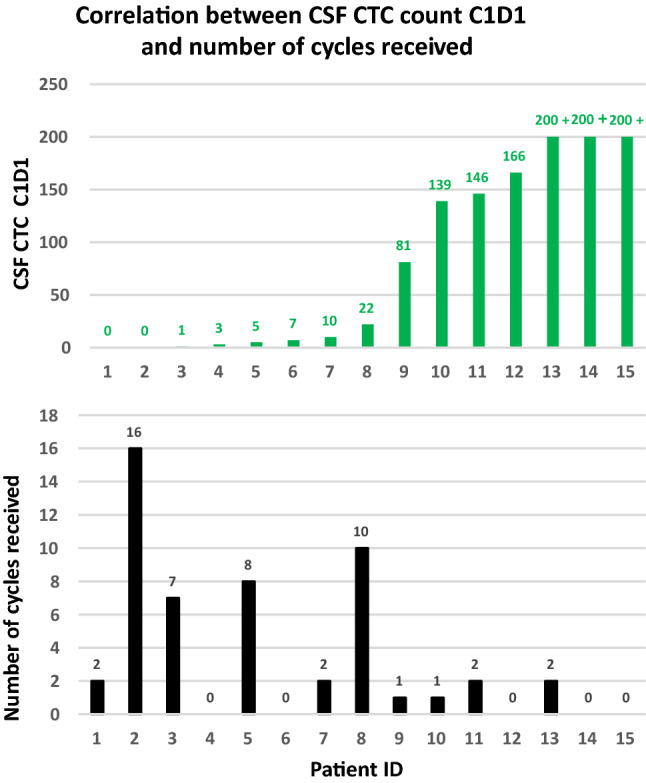
Correlations between CSF CTC enumeration cycle 1 day 1 and number of treatment cycles patients received. Numbers of CSF CTCs prior to treatment (upper panel) showing some trend of low CSF CTCs enumeration and longer duration of treatment (low panel)

Ten of 15 patients completed one or more cycles of treatment and had serial CSF CTC assessments (Fig. 2). Five out of 15 patients came off study before they completed cycle 1; due to clinical POD in 3, radiographic POD in spine in one and adverse side effect in one. In those 4out of 10 patients who received 7 and more cycles of therapy, median CSF CTC was 3 CTC/3 ml (range 0–22) prior to initiation of treatment; 6 out of 10 patients who stayed on study for only 1 or 2 cycles had median pre-treatment CSF of 110 CTC/3 ml (range 0–200+).

**Fig. 3:**
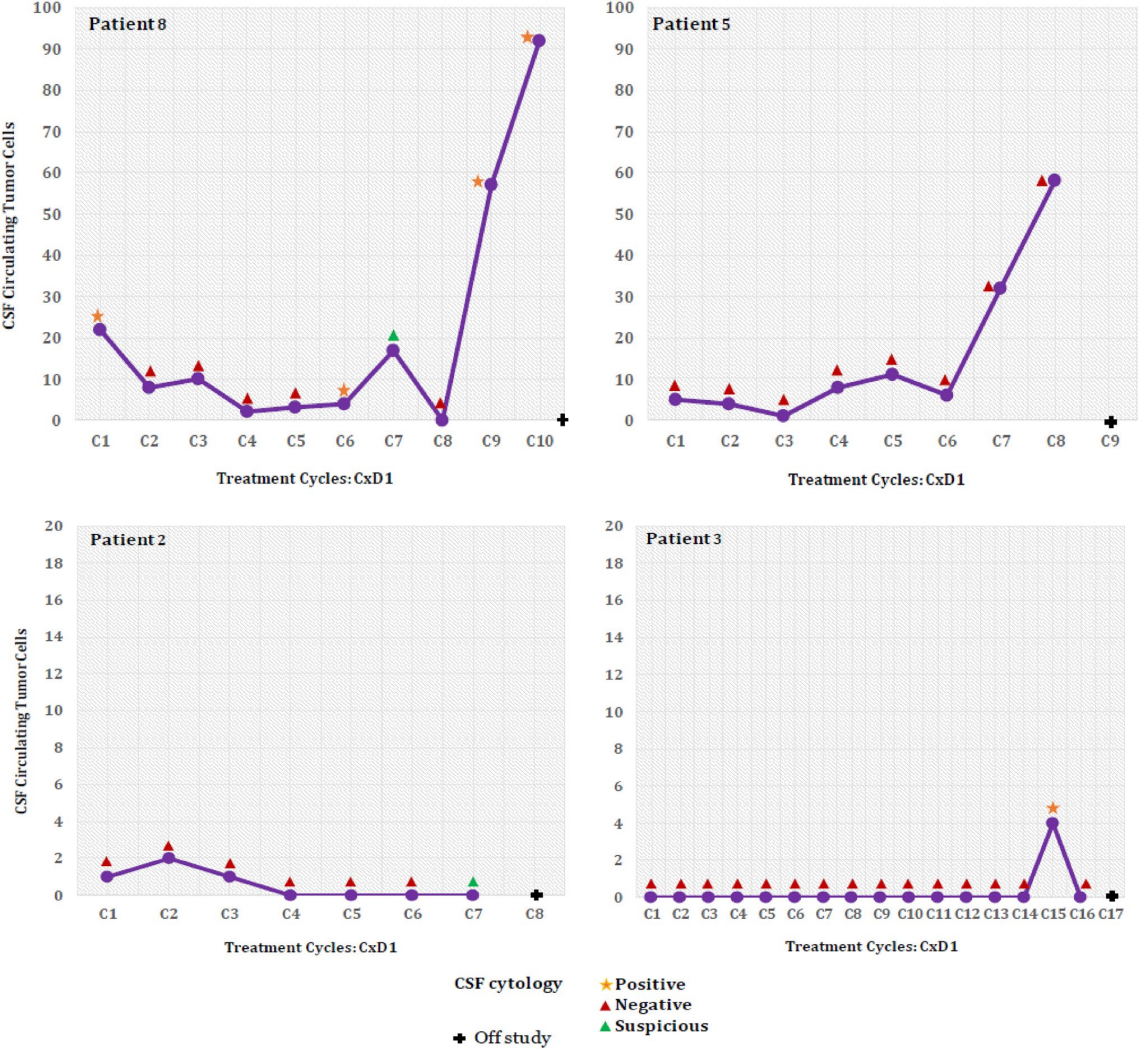
Changes in CSF CTC enumeration and CSF cytology in four patients with low baseline CSF CTCs who received > 7 cycles of IT therapy. The graphs illustrate the changes in CSF CTCs and CSF cytology overtime while patients continued treatment on a study. In patients 3, 5 and 8, a spike in CSF CTCs enumeration was noted 4–8 weeks prior to disease progression seen on MRIs

Four out of eight patients with low CSF CTC enumeration at baseline (range 0–22 CSF CTCs/3 ml) received ≥ 7 cycles of IT trastuzumab (Fig. 3). In 3 of those 4 patients, a decrease in CSF CTCs was observed during the first 2 months of treatment while some improvement was also seen on imaging and clinically. However, in the 4–8 weeks prior to definitive radiographic POD, CSF CTC count was going up in 3 patients, while CSF cytology remained negative in one (patient 5) and turned from negative to positive in 2 other patients. The remaining 6 patients stayed on study for a shorter duration: 2/6 (33%) completed 1 cycle, while 4/6 (67%) completed 2 cycles of treatment and then came off trial because of disease progression (Supplementary Fig. 1). In 50% of these patients (patients 9, 10, and 13) CSF CTCs numbers increased along with disease progression on neuroimaging**.** In 2 patients (patients 7 and 11) a decrease in CSF CTCs count at the time of disease progression was observed, while no CSF CTCs were detected in another patient (patient 1).

## Discussion

This is the largest study incorporating serial longitudinal enumeration of CSF CTCs in patients with HER2+ cancers and LM enrolled on a clinical trial. In this multicenter trial, we obtained CSF for CTCs analysis from multiple participating institutions prior to the initiation of LM-directed treatment, during treatment, and at progression. We compared these samples to CSF cytology samples collected at the same time, as well as MRI scans performed within a few days of CSF collection. Our study demonstrates CSF CTC analysis can easily be incorporated into multi-center prospective studies for patients with LM, given that the CellSave preservation tubes allow the specimen to be stored for up to 72 h and that CSF samples can be shipped unfrozen.

CSF CTC enumeration by the CellSearch® platform most likely reflects the overall disease burden in the CNS compartment, which can be easily calculated per 1 ml of CSF. We observed that patients with a higher CSF CTC enumeration were more likely to progress rapidly, suggesting a high burden of disease in the CSF compartment, which is not necessarily seen on neuroimaging. High disease burden in the CNS compartment at the time of diagnosis could correspond with the dismal overall survival of patients with LM, which is typically 3–4 months [2, 20–22]. Conversely, there was a group of patients with low CSF CTC enumeration prior to treatment who received 7 or more cycles of therapy before further POD of LM seen on neuroimaging, suggesting that CSF CTCs may serve as a prognostic biomarker or a biomarker of treatment response. Other important findings of this study were an observed drop in CSF CTC count in patients who responded to therapy, and a sudden spike in CSF CTC count 4–8 weeks prior to clinical or neuroimaging POD. Based on these results, we hypothesize that changes in CSF CTC enumeration might reflect the dynamic changes of LM disease before changes on MRI or CSF cytology are identified.

CSF CTC analysis was optional in this study and not all patients elected to participate, which is a limitation of this and many other studies. In addition, our cohort included patients with both recurrent and newly diagnosed LM with median time between LM diagnosis and the date of enrollment of 250 days. As a result, each group is relatively small and thus not amenable for correlations with overall survival and progression free survival. Another limitation of this study is that some of patients had LM in the brain and spine, however all CSF samples were collected only from an Ommaya reservoir and not via a lumbar puncture which could increase the change of identifying higher CTC count than from an Ommaya reservoir [11]. Regardless, this study features the largest number of patients in a clinical trial that explores the value of CSF CTCs as a biomarker at multiple time points during treatment. Our study suggests lower CSF CTC counts could represent lower disease burden in the CNS compartment at early stages of LM and the CSF CTC analysis could be incorporated in response assessment for patients with LM. In the future, it may be worthwhile to consider stratifying patients with LM based on CSF CTC count at time of diagnosis in clinical trials.

The ability to assess CSF CTCs for molecular markers [18] such as HER2/*neu* expression using a specific Her2/*neu* antibody phenotyping marker reagent is an important advantage to confirm that all cells in the CNS compartment expressed HER2/*neu* prior to initiation of treatment. This enables real-time confirmation of HER2/*neu* expression or loss of HER2/*neu* over time, which has been reported previously at other sites of metastases [23]. In our study, HER2/*neu* expression of CSF CTC was identified in 6 of 8 (75%) of patients at trial entry when CSF CTCs were detected. HER2/*neu* expression of CSF CTC was not identified in 2 patients: 1 with only a single CSF CTC, and another who had 81 CSF CTCs/3 ml. While the reason for lack of HER2/*neu* positivity in the last patient is not clear, we hypothesize that this could be due either to HER2/*neu* loss in the CSF during molecular evolution of cancer, or it represents a false positive HER2/*neu* FISH analysis of the archival tissue. Incorporation of molecular analysis of CSF performed by next generation sequencing analysis could answer this question.

Many oncologists struggle with the decision to place an Ommaya reservoir and recommend IT chemotherapy because some patients decline so rapidly. Our data suggests that CSF CTC enumeration may aid in the detection of LM at an earlier time and may quantify disease burden. The information gained from quantitating CSF CTCs at baseline may help guide these decisions once future, larger prospective trials have further established the role of CSF CTC enumeration to determine disease burden in the CSF compartment.

## Conclusion

In this study, we demonstrate the value of CSF CTC enumeration in patients with LM enrolled on a phase I/II dose escalation trial of intrathecal trastuzumab. Beyond standard EpCAM-based CTC analysis, we have employed HER2/neu expression analysis of CTCs using a specific Her2/neu antibody phenotyping marker reagent to confirm that pre-treatment CSF CTCs express HER2+. Our findings demonstrate that enumeration of CSF CTCs is a more accurate measurement of tumor burden in the CNS than standard CSF cytology and can be used as a biomarker of treatment response. CSF CTC analysis might be incorporated in clinical trials and in the clinical care of patients with LM from epithelial cancers.

## Electronic supplementary material

Below is the link to the electronic supplementary material.

Supplementary file1 (DOCX 337 kb)—Supplementary Fig. 1 Changes in CSF CTCs over time in patients who remains on study for ashort period of time

Supplementary file2 (DOCX 15 kb)

## Data Availability

The datasets used and analyzed during the current study are available from the corresponding author on reasonable request.
