# Supplementary figures and images for: Cerebrospinal fluid circulating tumor cells as a quantifiable measurement of leptomeningeal metastases in patients with HER2 positive cancer
Source: J Neurooncol. 2020 Jun 6;148(3):599–606. doi: 10.1007/s11060-020-03555-z (PMC7438284; doi:10.1007/s11060-020-03555-z)

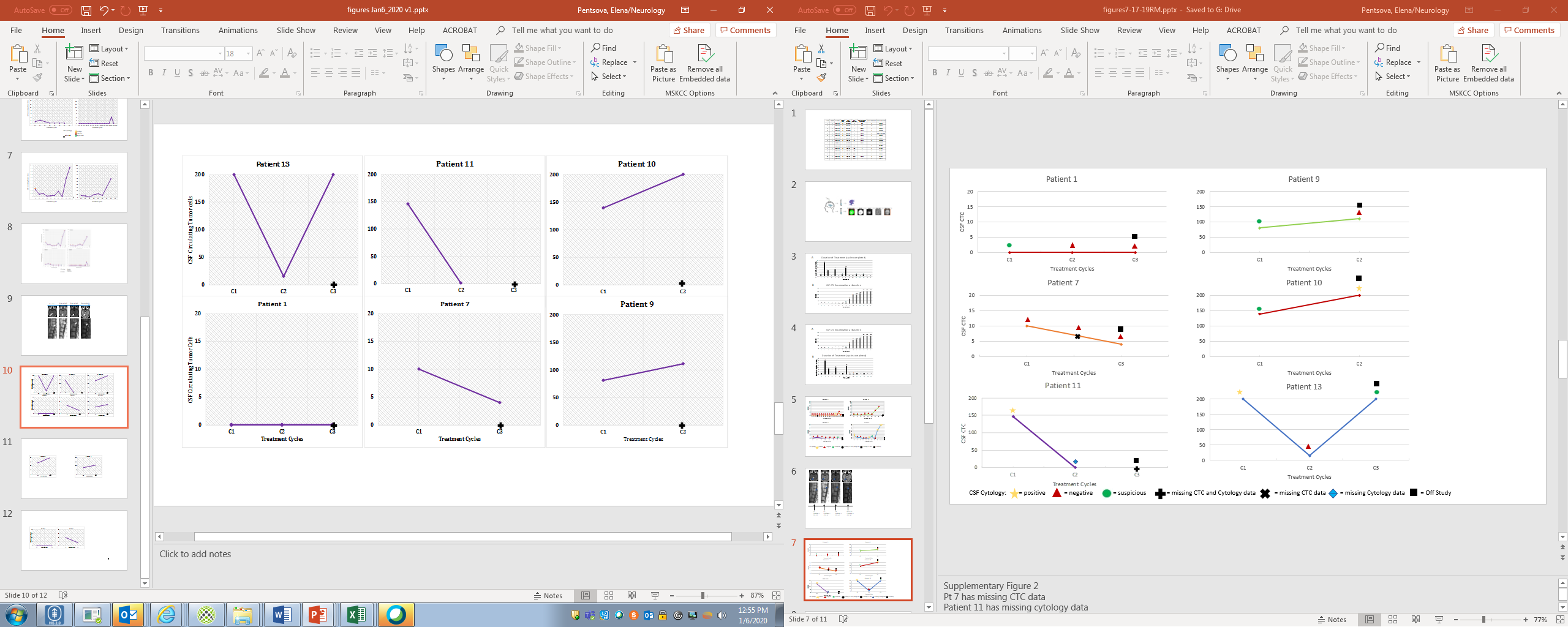
**Supplementary Figure**

Supplement: Supplementary file 1 — Supplementary file1 (DOCX 337 kb)—Supplementary Fig. 1 Changes in CSF CTCs over time in patients who remains on study for ashort period of time [file 11060_2020_3555_MOESM1_ESM.docx]
