# Supplementary material for: Cerebrospinal fluid circulating tumor cells as a quantifiable measurement of leptomeningeal metastases in patients with HER2 positive cancer
Source: J Neurooncol. 2020 Jun 6;148(3):599–606. doi: 10.1007/s11060-020-03555-z (PMC7438284; doi:10.1007/s11060-020-03555-z)
Supplement: Supplementary file 2 — Supplementary file2 (DOCX 15 kb) [file 11060_2020_3555_MOESM2_ESM.docx]

**Additional Supplementary Materials**

*Inclusion Criteria:*

- Confirmation of HER2 positivity. Patients may be IHC3+ and/or FISH positive; IHC 2+ HER2 patients are eligible with reflex FISH positive testing with a ratio ≥ 2.0
- Patients can have concomitant brain metastases as long as they do not require active treatment or have been treated
- CSF sampling required to document LM if not documented by MRI
- Life expectancy ≥ 8 weeks
- Normal renal (creatinine < 1.5 x upper limit of normal; liver (bilirubin ≤ upper limit of normal, transaminases ≤ 3.0 x upper limit of normal, except in known hepatic disease, wherein may be ≤ 5 x upper limit of normal) and blood counts (WBC ≥ 2.5, Neutrophils ≥ 1500, platelets ≥ 75,000, Hemoglobin ≥ 9).
- Left ventricular ejection fraction > 45%
- Karnofsky performance status ≥ 50
- Age ≥ 18 years
- Patients should be >24 hours from radiation therapy treatment to areas of the neuro-axis and all effects of treatment should have resolved
- Patients with a recent surgery should have recovered from all effects of the surgery and be cleared by their surgeon
- There is no limit on prior systemic or intrathecal therapies
- Must be willing to have an Ommaya reservoir placed and a candidate for an Ommaya reservoir placement
- Women of childbearing potential and sexually active males must commit to the use of effective contraception while on study
- Ability to sign an informed consent; can be signed by family member or health care proxy. Informed consent must be done prior to registration on study
- All patients must have signed, informed consent prior to registration on study

*Exclusion Criteria:*

- Cannot be on systemic agents (chemotherapy) that have CNS penetration (Temozolomide, BCNU, CCNU, Etoposide, Xeloda, Carboplatin, Navelbine, bevacizumab, CPT-11 and topotecan. Note: Other agents to be discussed with study PI) unless they have develop or have progressive or persistent leptomeningeal metastases while on the agent(s) and have controlled systemic disease
  - Note: May continue on IV trastuzumab, pertuzumab, TDM-1, lapatinib or hormonal agents if controlling systemic disease and developed LM while on therapy
  - Note: Patients requiring agents (such as those listed above) are eligible but will not be able too start treatment until after the first assessment by imaging and cytology
- Concurrent external beam radiation is not allowed with the exception of palliative radiotherapy to a localized region for pain control (i.e. vertebral disease, pelvis, etc) which is allowed while on the study protocol
  - Note: patients may need a CSF flow study at the discretion of the treating principal investigator. If a spinal block is seen by CSF flow study or MRI, it will need local RT prior to treatment.
- Patients who have controlled or responding leptomeningeal disease and develop brain metastases can remain on trial if their disease can be controlled with radiosurgery and does not require WBRT.
- No history of any other concomitant cancer (except non-melanoma skin cancer or carcinoma in-situ of the cervix) unless in complete remission and off all therapy for the disease for a minimum of 3 years.
- Patients should have no significant medical or psychiatric illness that would interfere with compliance and ability to tolerate treatment as outlined in the protocol.
- Women may not be pregnant or breast-feeding
- No known hypersensitivity to trastuzumab
